# Reliability Study of the Items of the Alberta Infant Motor Scale (AIMS) Using Kappa Analysis

**DOI:** 10.3390/ijerph19031767

**Published:** 2022-02-04

**Authors:** Jooyeon Ko, Hyun Kyoon Lim

**Affiliations:** 1 Department of Physical Therapy, Daegu Health College, Daegu 41453, Korea; 2 Safety Measurement Institute, Korea Research Institutes of Standards and Science, Daejeon 34113, Korea

**Keywords:** Alberta Infant Motor Scale (AIMS), reliability test, Fleiss’ kappa, preterm infants, delayed development

## Abstract

Purpose: We evaluated the interrater and intrarater reliabilities of the Korean version of the Alberta Infant Motor Scale (K-AIMS). Methods: For the interrater reliability test, six raters participated in the K-AIMS evaluation using video clips of 70 infants (aged between 0 and 18 months). One rater participated in an intrarater reliability test. Among 70 infants, 46 were born preterm and 24 were born full term. A total of 58 AIMS items were evaluated for supine, prone, sitting, and standing positions. A reliability analysis was conducted using ICC and Fleiss’ kappa. Results: The highest Fleiss’ kappa was found for the 4–7 months group for sitting (K = 0.701–1.000) and standing (K = 0.721–1.000), while the lowest K was the 3 months or under group for standing (K = 0.153–1.000). We found higher Fleiss’ kappa statistics when all infants were evaluated without grouping for the three positions (K = 0.727–1.000), except standing (K = 0.192–1.000), for the interrater analysis. Conclusion: Our results demonstrate the good reliability for the Korean version of the AIMS for Korean infants (preterm and full term).

## 1. Introduction

Preterm infants are babies defined as born under 37 weeks. The incidence rate of preterm delivery is estimated to be between 5% and 18% worldwide [1]. Children born preterm often demonstrate significantly poorer motor functions, cognitive outcomes, and language skills during development than those born full term [2]. Preterm infants often demonstrate atypical postures and movements, frequently manifesting as a hyper-extended neck and trunk, because they lack active flexor power compared to full-term infants [3,4]. Of note, the alignment of a preterm neonate’s musculoskeletal system while in neonatal intensive care plays a crucial role in determining their later postural shape and motor control [5,6]. In preterm infants, muscle tone, head control, and motor control development, including the lower and upper extremities, can be significantly less developed until the 18 corrected months [7]. Therefore, developmental process monitoring is highly recommended, i.e., performing a motor developmental evaluation periodically at the corrected full term, third month, sixth month, ninth month, twelfth month, and eighteenth month [8,9].

Regularly administered tests are valuable for detecting developmental disorders and predicting and preparing for further developments in preterm infants [10,11]. If administered within an appropriate timeframe, the infant motor development test could measure general motor performance, help both clinicians and parents make a more effective treatment plan and intervention strategy, and monitor motor development to predict neurodevelopmental outcome [4,12].

One of the infant motor development evaluation tools used most widely and recently is the Alberta Infant Motor Scale (AIMS) [13]. The AIMS evaluates 58 gross motor skills in supine, sitting, and standing positions. The AIMS could be used from 7 days (corrected age (CA)) to 18 months of age to monitor motor development [14]. The AIMS has been used to define, predict, and classify the level of motor development of preterm infants [15,16]. In one study, at eight months CA, only 56% of the preterm infants could sit briefly with no arm support, while over 90% of the term infants could perform the task [17]. The AIMS demonstrated its assessment validity for preterm infants’ movement quality [18]. Additionally, the AIMS can detect the imbalance between flexor and extensor muscle roles in the trunk and the lack of rotation in movements [19]. The postural control differences between preterm and full-term infants [20], and their gross motor trajectories [21], were also evaluated with the AIMS.

The AIMS was developed and standardized using 2,202 infants’ data from one week to 18 months of age in Alberta, Canada, in 1994 [14], and is used by many countries, including Brazil [22], Spain [23], China [24], Taiwan [25], and Greece [26]. Interrater reliability, consistency, and intra-class reliability were also assessed for each language by researchers in each country [1,27,28]. The AIMS evaluates qualitative movements, such as segment posture, weight-bearing, and anti-gravity movement as well as quantitative performance. It uses a motor development window that identifies a fully developed motor skill versus a newly developing motor skill. In this study, we evaluated the interrater and intrarater reliabilities of the Korean version of the AIMS (K-AIMS) for each item, subtotal, total scores with six raters using video clips of 70 infants using ICC and kappa statistics.

## 2. Materials and Methods

### 2.1. Infant Subjects

We recruited 70 healthy infants and parent volunteers using a social networking service, including 46 preterm infants under 18 months (CA), from three cities from 2017 to 2018. All infants and parents were Korean and monolingual. All parents signed consent forms before participation (IRB no. 2-1040781-AB-N-01-2017101HR). We excluded infants with congenital anomalies, acute illnesses, musculoskeletal disorders (fracture, peripheral neuropathy, and muscular system infection), and intraventricular hemorrhages of grades 3 and 4. The general characteristics of the infant subjects are summarized in Table 1.

### 2.2. Raters

Six physical therapists (raters A, B, C, D, E, and F) participated in the study for the interrater and intrarater reliability tests of K-AIMS. Rater A had under one year’s experience in child evaluation and pediatric physical therapy, and the others had 3 to 10 years’ experience. No rater had utilized the AIMS before participating in this study.

### 2.3. Evaluation Tool (AIMS)

The AIMS evaluates 58 items for four basic functional positions: 21 items for supine, 9 for prone, 12 for sitting, and 16 for standing. We did not intervene in infants’ performance, but observed their natural posture and movements during daily activities. Observers only changed body position when an infant could not change the position itself. The total test time was 20 min. All items were observed considering weight-bearing and anti-gravity movements. One point was given to an infant who showed the item defined by the AIMS component for a certain posture, and zero points were given when the item was not observed. Additionally, zero points were given to an item located within a motor development window, but which was not observed. Note that we did not modify the original AIMS at all, because AIMS could be translated and adapted in Korean well.

AIMS raw scores ranged from 0 to 58. The raw score was then converted into a percentile rank. This percentage was used for the parents’ and clinicians’ easy understanding, because they are accustomed to percentiles for anthropometric data, such as height, weight, and head circumference. A higher score represents more well-developed gross motor skills, while a lower score represents undeveloped gross motor skills. Infants scoring under the fifth percentile are at high risk for developmental differences [14].

### 2.4. Interrater and Intrarater Reliability Analysis

All raters had an education session (four hours) regarding the AIMS, including motor development theory and setting the motor development window. They could only begin participating in the main evaluation test when they demonstrated a higher than 90% interrater correlation during the preliminary training session. We did not use the same video clips in the preliminary training sessions and the main test. For the main reliability evaluation, raters completed AIMS evaluations of 70 video clips, recorded under standard conditions. One of the authors exclusively performed the AIMS administration in a spacious and comfortable room in the presence of parents. One assistant PT recorded the whole process for the reliability scoring. Four different positions (supine, prone, sitting, and standing) were prepared from the recording after eliminating portions of the video unnecessary for the AIMS reliability test. The six raters being evaluated scored the infants on the AIMS by watching video recordings of 70 infants in four positions in a training room. To avoid potential bias and ensure independent scoring, raters were not allowed to exchange opinions on the tested findings. Video recordings were played three times each, and one additional play was allowed per the rater’s request. For the intrarater reliability test (rater A), the AIMS tests were repeated four weeks apart with the same video clips [25].

### 2.5. Data Analysis

We divided all infants’ data into three groups: 0–3 months, 4–7 months, and 8 months or over. These age groups consisted of preterm infants’ corrected ages (CA) and full-term infants’ chronological ages. For instance, a prematurely delivered infants’ chronological age (or age from date of birth) may be nine months, but if its corrected age (or age from original due date) was seven months old, it was included in the 4–7 months group.

Total AIMS scores per position were analyzed statistically. Interrater reliability among the six raters (A, B, C, D, E, and F) and intrarater reliability by rater A for evaluations conducted four weeks apart were analyzed using Fleiss’ kappa analysis and the intraclass correlation coefficient (ICC) at a 95% confidence interval. We used a Bland–Altman plot to assess the intrarater reliability of the AIMS total score. In Fleiss’ kappa analysis, the following definitions were used: 0 = no agreement, 0.1–0.19 = poor agreement, 0.20–0.39 = fair agreement, 0.40–0.59 = moderate agreement, 0.60–0.79 = substantial agreement, and 0.80–1.00 = almost perfect agreement [29]. ICCs were interpreted as excellent, good, moderate, and poor for >0.90, 0.75–0.90, 0.50–0.75, and <0.5, respectively [30]. We used IBM SPSS Statistics 27 at a significance level of 0.05.

## 3. Results

### 3.1. Interrater and Intrarater Reliability of AIMS for Each Item

For the interrater reliability, Six raters showed Fleiss’ kappa (K) statistics ranging from 0.153 to 1.0 for four positions. In general, the highest Fleiss’ kappa was found for the 4–7 months group for sitting (K = 0.701–1.000) and standing (K = 0.721–1.000), while the lowest K was from the 3 months or under group (K = 0.153–1.000) for the standing position. We found higher Fleiss’ kappa when all infants were evaluated without grouping for three positions (K = 0.727–1.000), except standing (K = 0.192–1.000). See the summaries in Table 2 for further detail.

For the intrarater reliability, rater A showed Fleiss’ kappa (K) statistics ranging from 0.250 to 1.0 for four positions. See the summaries in Table 3 for further detail.

### 3.2. Interrater and Intrarater Reliability of AIMS for Subtotal and Total Scores

ICC analysis results from the six raters for each position and the total score were 0.80 to 1.00 (AVE. = 0.97, STD = 0.05) for the interrater reliability (Table 4). The minimum ICC (=0.796) was found for standing position for infants 3 months or under. Otherwise, all ICCs were greater than 0.96 for interrater reliability.

**Figure 1:**
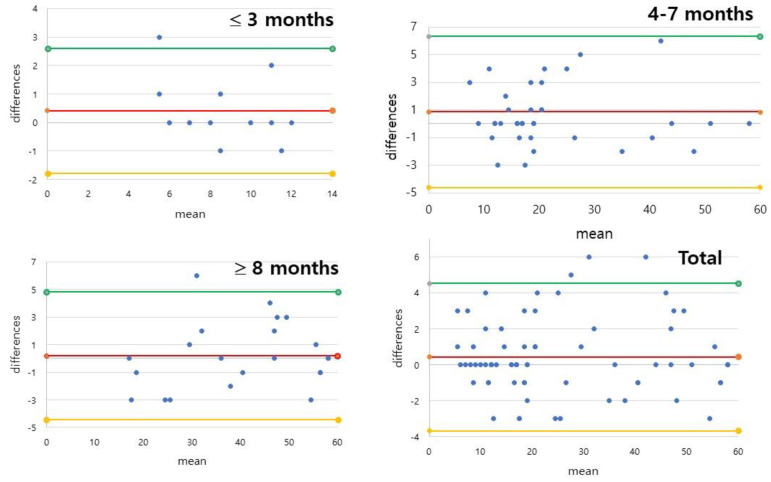
Bland–Altman plots of intrarater reliability of the AIMS (Alberta Infant Motor Scale) total scores for rater A. In summary, the average difference and SD for all infants recruited in this study were 0.43 and 2.09, with 4.53 and −3.68 for the upper and lower limits.

ICC analysis results from rater A for each position and the total score were 0.75 to 1.00 (AVE = 0.93, STD = 0.08) for intrarater reliability. An ICC of less than 0.8 was only found for the supine (0.749) and sitting positions (0.776) in the 3 months or under group. All other ICCs were greater than 0.85. The Bland–Altman analysis for total score gave the average difference of 0.42 and a standard deviation (SD) of 1.11, with 2.60 and −1.77 for the upper and lower limits, for the infants 3 months or under (Figure 1). The average difference and SD were 0.86 and 2.79, with 6.33 and −4.61 for the upper and lower limits, for the 4–7 months infants. The average difference and SD were 0.18 and 2.37, with 4.82 and −4.46 for the upper and lower limits, for the infants older than 8 months. In summary, the total average difference and SD for all infants recruited in this study were 0.43 and 2.09, with 4.53 and −3.68 for the upper and lower limits.

## 4. Discussion

We evaluated the interrater and intrarater reliability of the Korean version of the AIMS using six raters. Among them, one rater’s repeated evaluation results were analyzed for intrarater reliability. For the study, we used video recordings from 70 infants in four positions (prone, supine, sitting, and standing). Fleiss’ kappa values showed highly acceptable agreement in infants with a corrected or chronological age of 3 months or more. In addition, the ICC values of interrater and intrarater reliabilities for the subscales and total scores of K-AIMS were good to excellent.

Reliability, consistency, and stability studies of a newly introduced scale are fundamental prerequisites before its implementation [28,31]. The AIMS has been studied for its improved ability to detect delayed motor development in preterm infants [32] compared to other existing previous scales, such as the Bayley-III [4,18]. The AIMS’s interrater reliability has also been studied [31]. Of note, the AIMS has demonstrated moderate to strong correlation (r = 0.78–0.9) with the Bayley Motor Scale [25].

The AIMS has also been translated into many languages and studied for its reliability in many countries, including Taiwan [25], China [24], Thailand [33], Serbia [28], Brazil [22], and Japan [34]. It also underwent cross-country validation with Brazilian infants [35], and norm comparison between Canadian and Turkish infants [13] and Dutch and Canadian infants [36]. As language and cultural context may affect the AIMS’s validity, reliability tests should be conducted after translation [37], because they may elucidate significant differences between the cultural context and normative sample [38]. This study was, therefore, necessary, because reliability studies on the AIMS translated into Korean had not yet been conducted.

Reliability tests made with intraclass correlation coefficients (ICCs) demonstrated high ICC values (0.97–0.99) for Taiwanese infants [25], Chinese high-risk infants (0.81–0.99) [24], and Serbian infants (ICCs ≥ 0.75), except for the standing position in the 4–7 months group [28]. Our results also exhibited similarly high ICCs > 0.9 (AVE = 0.93, STD = 0.08) for most of the positions, except the supine and sitting positions in infants 3 months or under old. Additionally, interrater comparisons were also within a reliable range (ICCs = 0.98–0.99) [24], except for infants 3 months or under, and 4–7 months for the standing position (0.73 and 0.75 each) [25]. 

Many previous studies performed multi-rater reliability tests using ICC but not kappa. Fleiss’ kappa test (the reliability test for six interrater tests) exhibited better than substantial agreement (98.3%, 91.4%) for the 4–7 months and ≥8 months groups for 50 AIMS items (AVE = 94.9%). We also found lower reliability for infants 3 months or under, as previous studies have found [14,24]. The lowest reliability was found for supported standing (item 2) in standing subscale for infants 3 months or under, because infants could not perform this evaluation item. Regardless of infant age, interrater reliability showed moderate to substantial agreement for forearm support (item 6) in prone subscale. Pull to sit (item 3; 3 months or under) and sitting to prone (item 10; 8 months or over) from the sitting subscale showed low interrater reliability in this study. No significant difference between novice and experienced PT was found in our study. In the similar study using a Japanese population [34], the raters were also non-expert PTs and expert PTs, like in this study, and the ICC results for the interrater and intrarater reliabilities were good to high, which are similar results in this study.

We found the lowest Fleiss’ kappa (=0.25) for prone mobility (item 5) in prone subscale in the intrarater analysis of 0–3 months old infants, meaning that this is the most challenging infant age for which to evaluate motor control. We found more than substantial agreement (>90%) from the 4–7 months and ≥8 months groups. The AIMS gave a higher Fleiss’ kappa when infants were older. Live observations or video recordings [39,40] were used for the reliability test on motor skills. No significant difference was observed between the live observation and video recordings [41]. We used video clips for the K-AIMS assessment for convenience.

## 5. Conclusions

Our study demonstrates that for each K-AIMS item’s score, subscales and total scores are reasonably reliable when screening for motor development delay and monitoring infants’ progress in South Korea.

## 6. Limitations

We could not fully investigate sensitivity to temporal variations, which should be evaluated and compared for the same infant with an appropriate time gap to understand motor development.

## Figures and Tables

**Table 1:** General characteristics of the subjects (*n* = 70).

Variables	
Age (days)	
Preterm infants (CA)	145.2 ± 92.8 (16–431)
Full-term infants	276.3 ± 103.4 (180–540)
Gestational age (days)	
Preterm infants	209.5 ± 48.5 (161–258)
Full-term infants	273 ± 7 (266–280)
Birth weight (grams)	
Preterm infants	1790 ± 1230 (560–3020)
Full-term infants	2885 ± 157.5 (2500–3810)
Preterm vs. full term (numbers)	
Preterm infants	46 (65.7%)
Full-term infants	24 (34.3%)
Gender	
Girls	21 (30.0%)
Boys	49 (70.0%)
Age bands (months)	
3≥	12 (17.1%)
4–7	36 (51.4%)
8≤	22 (31.4%)

Unit: Mean ± standard deviation or *n* (%), CA: corrected age.

**Table 2:** Interrater reliability of the items of the AIMS (Alberta Infant Motor Scale) among six raters using Fleiss’ kappa.

	≤3 Months (*n* = 12)				4–7 Months (*n* = 36)				≥8 Months (*n* = 22)				Total (*n* = 70)			
Items	Prone	Supine	Sitting	Standing	Prone	Supine	Sitting	Standing	Prone	Supine	Sitting	Standing	Prone	Supine	Sitting	Standing
1	1.000	1.000	1.000	1.000	1.000	1.000	1.000	1.000	1.000	1.000	1.000	1.000	1.000	1.000	1.000	1.000
2	0.775	1.000	1.000	0.153	1.000	1.000	0.897	1.000	1.000	1.000	1.000	1.000	0.796	1.000	0.958	0.192
3	1.000	0.649	0.635	-	0.792	1.000	0.701	0.721	1.000	1.000	1.000	0.801	0.946	0.808	0.823	0.830
4	0.831	0.757	-	-	0.881	0.721	0.779	1.000	1.000	0.587	1.000	0.942	0.906	0.822	0.887	0.979
5	0.738	0.531	-	-	0.727	0.726	0.745	1.000	1.000	0.832	0.915	0.866	0.830	0.778	0.861	0.945
6	0.675	0.871	-	-	0.583	0.693	0.862	0.949	0.441	0.913	0.814	0.764	0.727	0.809	0.894	0.871
7	1.000	0.775	-	-	0.707	0.790	0.919	0.860	0.842	0.735	0.947	0.825	0.821	0.830	0.955	0.859
8	1.000	0.814	-	-	0.797	0.755	0.896	0.843	0.773	0.689	0.862	0.891	0.861	0.814	0.923	0.890
9	-	0.606	-	-	0.783	0.650	0.885	0.866	0.845	0.777	0.818	0.768	0.856	0.740	0.896	0.879
10	-		-	-	0.892		0.882	0.876	0.853		0.500	0.845	0.924		0.763	0.868
11	-		-	-	0.852		0.943	0.919	0.939		0.817	0.951	0.921		0.911	0.938
12	-		-	-	0.865		0.933	0.912	0.818		0.597	0.947	0.884		0.787	0.933
13	-			-	0.975			0.919	0.926			0.947	0.969			0.935
14	-			-	0.889			0.879	0.682			0.585	0.831			0.756
15	-			-	0.812			0.919	0.716			0.788	0.817			0.862
16	-			-	0.910			0.951	0.835			0.901	0.904			0.936
17	-				0.931				0.812				0.895			
18	-				0.920				0.737				0.851			
19	-				0.856				0.722				0.807			
20	-				0.793				0.662				0.749			
21	-				0.872				0.786				0.839			

**Table 3:** Intrarater reliability (rater A) of the items of the AIMS (Alberta Infant Motor Scale) using Fleiss’ kappa.

	≤3 Months (*n* = 12)				4–7 Months (*n* = 36)				≥8 Months (*n* = 22)				Total (*n* = 70)			
Items	Prone	Supine	Sitting	Standing	Prone	Supine	Sitting	Standing	Prone	Supine	Sitting	Standing	Prone	Supine	Sitting	Standing
1	1.000		0.308	1.000	1.000	1.000	0.654	1.000	1.000	1.000	0.478	1.000	1.000	1.000	0.478	1.000
2	0.400		1.000	0.400	1.000	1.000	0.769	1.000	0.485	1.000	0.864	0.735	0.485	1.000	0.864	0.735
3	0.824	0.400	1.000	-	0.654	0.654	0.769	0.862	0.818	0.569	0.899	0.850	0.818	0.569	0.899	0.850
4	0.750	0.625	-	-	0.842	0.800	0.780	1.000	0.906	0.881	0.886	0.964	0.906	0.881	0.886	0.964
5	0.250	0.625	-	-	0.520	0.833	0.625	1.000	0.639	0.827	0.745	0.925	0.639	0.827	0.745	0.925
6	1.000	1.000	-	-	0.536	0.667	0.799	0.842	0.730	0.714	0.909	0.811	0.730	0.714	0.909	0.811
7	1.000	-	-	-	0.827	0.942	0.852	0.636	0.857	0.886	0.909	0.598	0.857	0.886	0.909	0.598
8	1.000	-	-	-	1.000	0.778	1.000	0.873	0.881	0.885	0.969	0.881	0.881	0.885	0.969	0.881
9	-	-	-	-	0.630	0.869	0.545	1.000	0.706	0.910	0.766	0.906	0.706	0.910	0.766	0.906
10	-		-	-	0.862		1.000	0.471	0.882		0.868	0.618	0.882		0.868	0.618
11	-		-	-	0.862		0.893	1.000	0.942		0.964	0.925	0.942		0.964	0.925
12	-		-	-	0.916		0.842	1.000	0.902		0.811	0.841	0.902		0.811	0.841
13	-			-	1.000			1.000	1.000			0.925	1.000			0.925
14	-			-	0.801			1.000	0.851			0.639	0.851			0.639
15	-			-	0.722			1.000	0.711			0.747	0.711			0.747
16	-			-	0.906			1.000	0.930			0.785	0.930			0.785
17	-				1.000				0.850				0.850			
18	-				0.620				0.783				0.783			
19	-				0.640				0.742				0.742			
20	-				0.636				0.720				0.720			
21	-				0.786				0.883				0.883			

**Table 4:** Interrater (six raters) and intrarater (rater A) reliabilities of subtotal and total scores of the AIMS (Alberta Infant Motor Scale) using ICC and 95% CI.

	Interrater Reliability							
	≤3 Months (*n* = 12)		4–7 Months (*n* = 36)		≥8 Months (*n* = 22)		Total (*n* = 70)	
	ICC	95% CI	ICC	95% CI	ICC	95% CI	ICC	95% CI
Prone total	0.981	0.956–0.994	0.979	0.966–0.988	0.979	0.961–0.990	0.987	0.981–0.991
Supine total	0.963	0.917–0.988	0.981	0.969–0.989	0.977	0.959–0.989	0.985	0.979–0.990
Sitting total	0.962	0.916–0.988	0.993	0.989–0.996	0.992	0.985–0.996	0.996	0.994–0.997
Standing total	0.796	0.544–0.932	0.998	0.996–0.999	0.995	0.991–0.998	0.997	0.996–0.998
Total	0.940	0.849–0.984	0.998	0.996–0.999	0.995	0.991–0.998	0.998	0.997–0.999
	**Intrarater Reliability**							
	**≤3 months** **(*n* = 12)**		**4–7 months** **(*n* = 36)**		**≥8 months** **(*n* = 22)**		**Total** **(*n* = 70)**	
	**ICC**	**95% CI**	**ICC**	**95% CI**	**ICC**	**95% CI**	**ICC**	**95% CI**
Prone total	0.855	0.495–0.958	0.973	0.947–0.986	0.982	0.971–0.989	0.982	0.971–0.989
Supine total	0.749	0.129–0.928	0.885	0.775–0.942	0.940	0.904–0.963	0.940	0.904–0.963
Sitting total	0.776	0.187–0.933	0.971	0.944–0.985	0.987	0.979–0.992	0.987	0.979–0.992
Standing total	0.867	0.540–0.962	0.995	0.990–0.997	0.994	0.991–0.996	0.994	0.991–0.996
Total	0.940	0.792–0.983	0.988	0.976–0.994	0.994	0.991–0.996	0.994	0.991–0.996
